# Screening for novel enzymes from metagenome and SIGEX, as a way to improve it

**DOI:** 10.1186/1475-2859-4-8

**Published:** 2005-03-25

**Authors:** Jiae Yun, Sangryeol Ryu

**Affiliations:** 1 Department of Food and Animal Biotechnology, School of Agricultural Biotechnology, Seoul National University, Shillim-dong, Kwanak-gu, Seoul 151–742, Korea; 2 Center for Agricultural Biomaterials, Seoul National University, Shillim-dong, Kwanak-gu, Seoul 151–742, Korea

## Abstract

Metagenomics has been successfully applied to isolate novel biocatalysts from the uncultured microbiota in the environment. Two types of screening have been used to identify clones carrying desired traits from metagenomic libraries: function-based screening, and sequence-based screening. Both function- and sequence- based screening have individual advantages and disadvantages, and they have been applied successfully to discover biocatalysts from metagenome. However, both strategies are laborious and tedious because of the low frequency of screening hits. A recent paper introduced a high throughput screening strategy, termed substrate-induced gene-expression screening (SIGEX). SIGEX is designed to select the clones harboring catabolic genes induced by various substrates in concert with fluorescence activated cell sorting (FACS). This method was applied successfully to isolate aromatic hydrocarbon-induced genes from a metagenomic library. Although SIGEX has many limitations, it is expected to provide economic advantages, especially to industry.

## Review

More than 99% of bacteria in the environment cannot be cultured using conventional methods [1,2]. To study and use the genomes of such uncultured microbes, metagenomics has been in the spotlight since the 1990s [3]. Many studies have constructed metagenomic libraries to search for novel biocatalysts or molecules for biotechnological and pharmaceutical applications. To date, metagenomics has uncovered a variety of novel genes ranged from small genes conferring enzymes to complex gene clusters encoding proteins involved in antibiotic production, using different kinds of vectors such as plasmids, cosmids, fosmids and bacterial artificial chromosomes [4]. However, the efficiency of searching for novel catalysts from metagenome can still be improved. Screening for desired traits needs improvement because this step is still labor-intensive and time-consuming. This mini-review discusses the strategies that have been used in metagenome screening, particularly the recently introduced screening strategy, SIGEX. The characteristics of the discussed strategies are summarized in Table 1.

### Functional- and sequence based screening

Two strategies are generally used to screen and identify novel biocatalysts or genes involved in the production of antibiotic from metagenomic libraries: function-based and sequence-based screening. In function-based screening, clones expressing desired traits are selected from libraries, and aspects of molecular biology and biochemistry of active clones are analyzed. Many enzymes of industrial importance have been discovered using this strategy (Table 1). This approach enables the rapid acquisition of clones that have potential of direct application in industry. Moreover, this screening method can detect genes with completely novel DNA sequences, which may have functions distinct from known biocatalysts. However, function-based screening has several limitations. This method requires expression of the function of interest in the host cell (e.g. *Escherichia coli*) as well as clustering of all of the genes required for the function. In addition, efficient and economical screening methods for desired traits must be established to facilitate high-throughput-screening of vast libraries.

Conversely, sequence-based screening is not dependent on the expression of cloned genes in heterologous hosts. Generally it is based on the conserved DNA sequences of target genes. Hybridizations or PCR are performed based on the deduced DNA consensus. However the limitations of sequence-based screening are that DNA consensus must be analyzed and determined, which cannot be applied to many biocatalysts, and that it does not guarantee acquisition of full-length genes or full gene clusters that are necessary for the production of the desired product. Moreover, the sequence-based screening never screens desired genes with completely different sequences, and easy expression or correct folding of the screened gene is not assured. In metagenomics, several novel enzymes of industrial importance have been screened successfully using this strategy, but the typical application of sequence-based screening is to obtain ribosomal RNA genes for phylogenetic surveys.

As a form of sequence-based screening, shotgun sequencing of metagenomic libraries has recently provided vast amount of data, including phylogenetic relationships, millions of novel genes, and deduced metabolic pathways of uncultured bacteria [5-7]. Some of the novel genes might be of industrial importance. However, shotgun sequencing is extremely expensive and labor intensive, especially when one aims to discover genes of desired traits. Moreover, since the data from shotgun sequencing are analyzed in sequence-similarity searches based on constructed database, this method is not free from the limitations of sequence-based screening.

Although both of function- and sequence-based screening strategies have been applied to isolate novel biocatalysts from metagenome, both approaches are laborious due to the low frequency of clones with desired traits (e.g. 4 from 930,000) [8]. To improve the frequency of screening, several strategies have been developed. For example, to overcome the difficulties with the heterologous expression of secondary metabolites, *Streptomyces lividnas *or *Pseudomonas putida *have been used in addition to *E. coli *[9-11]. In addition, enrichment steps for uncultured microorganisms containing the desired traits have been used successfully before library construction [12-15]. This approach is also advantageous because it overcomes the cloning difficulties due to the contaminants in environmental samples. Yet, the biased selection of metagenome argues against enrichment.

### SIGEX, the third screening method

In an effort to improve the frequency of screening hits, Kazuya Watanabe and colleagues proposed substrate-induced gene expression screening (SIGEX), and its utility was evaluated for the screening of aromatic hydrocarbon-induced genes from a groundwater metagenome library [16].

**Figure 1:**
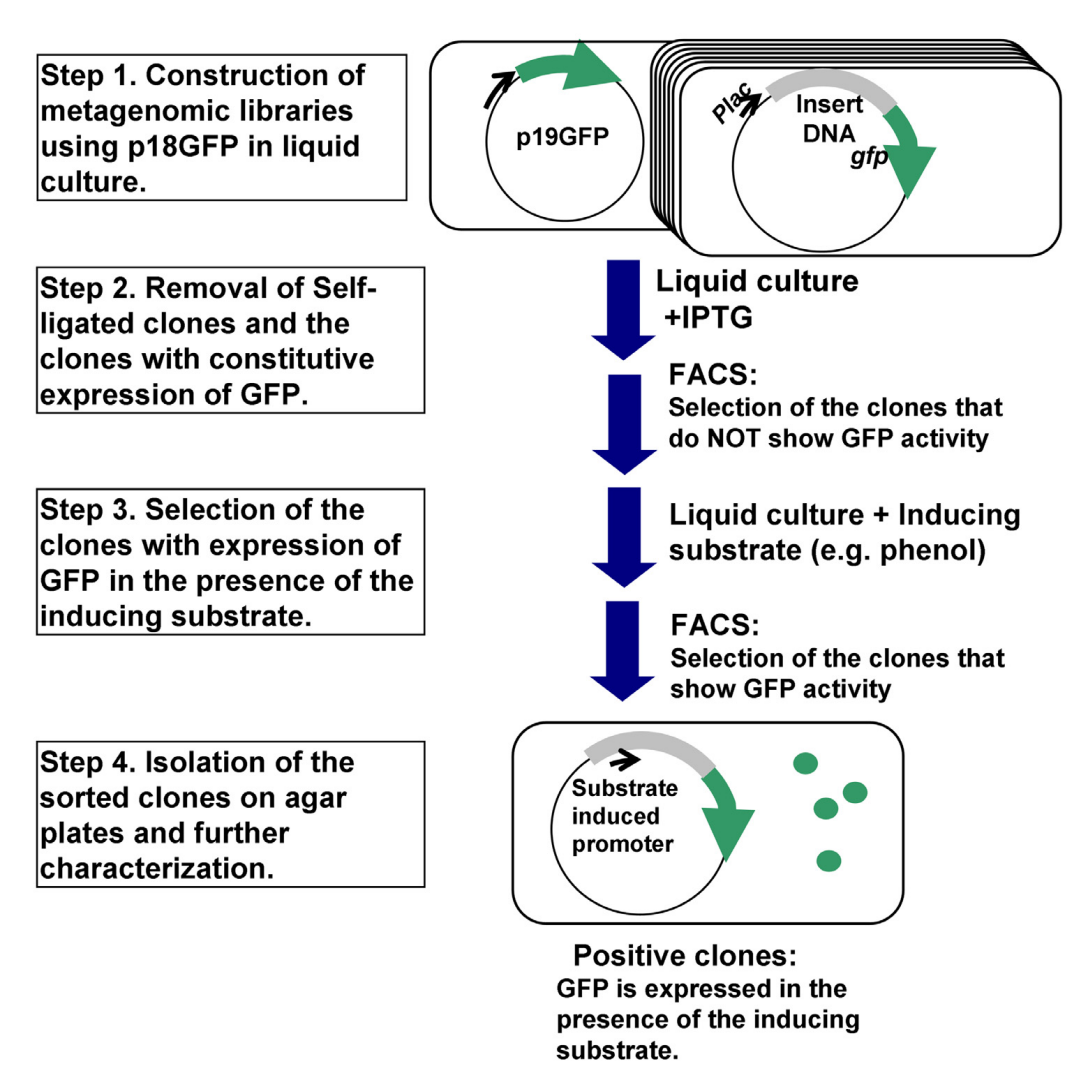
Schematic diagram of the SIGEX process

To design of SIGEX is based on the facts that the expression of catabolic genes is generally induced by substrates or metabolites of catabolic enzymes, and that the expression of catabolic genes is controlled by regulatory elements located proximately in many cases. SIGEX screens the clones harboring desired catabolic genes that are expressed in the presence of substrates but are not expressed in the absence of substrates. The procedure is described in Figure 1. To make SIGEX a high-throughput process, an operon-trap vector (p18GFP) was constructed, in which the cloning site divides the *lac *promoter and the *gfp *structural gene. Metagenomic libraries are constructed using p18GFP (Step 1). Self-ligated clones and the clones expressing *gfp *constitutively are removed by IPTG induction in the absence of the substrate (Step 2). The expression of catabolic genes in cloned metagenomic DNA is determined by *gfp *expression in the presence of the substrate (Step 3), and then the positive clones are separated on agar plates and characterized (Step 4). Fluorescence-activated cell sorting (FACS) is applied to the sorting and separation of GFP-expressing clones, *i.e*. the clones with desired catabolic genes.

Watenabe and colleagues constructed a metagenomic library using groundwater sample and successfully applied SIGEX to isolate 33 clones induced by benzoate and two clones induced by naphthalene from 152,000 clones [16]. In addition these researchers showed that enzyme Bzo71-8 P450 from the metagenomic library is novel. These data demonstrate the practice of SIGEX for screening catabolic genes using appropriate inducers or substrates, and the possibility of SIGEX to yield more active clones than conventional screening methods.

### Advantages and disadvantages of SIGEX

SIGEX has many advantages in metagenome screening. It provides an efficient and economic way of high throughput screening, because it allows for semi-automation thereby saving time, labor and expenses. This is particularly important for industrial applications. SIGEX is also advantageous because it can detect catabolic genes for which colorimetric or other on-plate screening methods are not established. Using this strategy, the Watanabe group screened hydrocarbon-induced genes, which are difficult to screen using conventional methods [16]. In addition, SIGEX does not require the modified substrates that are often used in colorimetric screenings, which are occasionally toxic, cause side-effects, and are generally more expensive than unmodified substrates. Moreover, SIGEX enables the deduction of the substrates for an unknown enzyme from the induction substrate used in the SIGEX screening. This helps to increase scientific knowledge about the genetics of previously unknown and hypothetical genes.

However, the application of SIGEX has limitations. First, SIGEX is sensitive to the structure and orientation of genes with desired traits. SIGEX misses catabolic genes that are expressed constitutively. In addition, SIGEX cannot detect any active clones in which the desired catabolic genes are cloned in the direction opposite *gfp*. Moreover, it misses the active clones that have a transcription terminator between catabolic genes and the following *gfp*. In these cases, conventional function-based screening methods have been successfully applied to detect active clones. Particularly for the last reason, SIGEX is not suitable for applying to metagenomic libraries harboring large insert DNA due to the abundance of transcription terminators [17]. Because the probability of finding a screening hit using function-based screening increases exponentially with DNA insert size [18], the application of SIGEX should be considered carefully, especially when large pieces of environmental DNA are readily prepared. Second, substrates that do not migrate to the cytoplasm cannot be used with SIGEX. Many enzymes, such as amylases, proteases, lipases, cellulases and xylanases target macromolecules that do not migrate to the cytoplasm. To date, such enzymes have been detected by the incidental natural secretion of intracellular proteins or artificial cell disruption. Since many of these enzymes are of industrial importance, this drawback cannot be overlooked. Finally, the gate setting in FACS and the media conditions containing the inducer are critical for discriminating false-positive and false-negative results. Therefore, when SIGEX is applied, these drawbacks should be considered carefully.

In conclusion, under the conditions where SIGEX is applicable, *i.e*. when appropriate substrates and target genes are selected, and the gate-setting of FACS is optimized, SIGEX can be a very powerful tool, especially to industry, for screening genes involved in antibiotics production or biodegradation induced by small molecules.

## Conclusion

Metagenomics has proven effective for isolating novel biocatalysts from the environment as well as to acquire ecological data. Its scale and scope have been expanded since its concept was first introduced. For example, robotic automation has been developed to construct and screen metagenomic libraries, and large corporations have provided substantial funding for metagenomics. However the major problems in constructing metagenomic libraries remain to be solved. No standard protocol exists for isolating sufficiently purified metagenomic DNA from environmental samples. In addition, the heterologous expression system of genes from metagenome requires further improvement. An efficient expression system other than *E.coli *should be developed and settled (trials have recently begun). Moreover, since the conventional screening system is costly and time-consuming despite the recent improvement of automation, it remains necessary to develop more effective and economic strategies. SIGEX is a good way to overcome this bottleneck. Therefore, to exploit the enormous genetic resources in the environment in more efficient ways, these problems should be solved, and improved technologies should be developed.

## Figures and Tables

**Table 1:** Comparison of the screening methods for metagenomeic libraries

	***Function-based screening***	***Sequence-based screening***	***SIGEX***
**Screening principle**	• Detecting changes by enzymatic reactions (e.g. halo formation around the colonies)	• PCR or Southern hybridization based on the DNA sequence consensus	• Trapping the operon induced by a substrate and sorting using FACS
**Advantages**	• Secures a complete form of gene or gene cluster required for desired traits • Potentially obtains completely novel genes.	• Overcomes the limitations of the heterologous expression	• Fast and economical • Any substrates that can be introduced into cytoplasm can be used in its native forms.
**Disadvantages**	• Must satisfy the expression conditions (transcription, translation, folding, secretion) in heterologous hosts	• Requires a database and analyses of the DNA sequence consensus. • Does not guarantee the acquisition of complete forms of genes or gene clusters.	• Sensitive to the orientation of the genes with desired traits • Cannot use substrates that do not migrate to cytoplasm • Sensitive to the initial FACS setting
**Examples**	antibiotics [9, 19-22], genes involved antibiotic resistance [9, 23, 24], agarases [15], amidases [13], amylases [15, 21, 25, 26], esterase/lipases [8, 15, 21, 27, 28], xylanases [29], 4-hydoxybutyrate dehydrogenase [30] alcohol oxidoreductases [14], pectate lyases [31]	amylases [26], polyketide synthases [32, 33]	Benzoate-degratative or catechol degradative operon, P450 enzyme [16]
